# Bridging self-control and prosocial behavior in early adolescents: a simulation-based node-perturbation analysis

**DOI:** 10.3389/fpsyg.2026.1774626

**Published:** 2026-06-17

**Authors:** Fang Yi, Zhi Zhang, Yilin Ren

**Affiliations:** 1 College of Physical Education and Health, Guangxi Normal University, Guilin, China; 2 Zhuhai Research Center for Women and Children's Sports Culture, College of Sports, Jinan University Zhuhai Campus, Zhuhai, China

**Keywords:** early adolescence, hypothesis-generating simulation, latent profile analysis, network analysis, node perturbation, prosocial behavior, self-control

## Abstract

**Background:**

Prosocial behaviors during early adolescence are closely related to self-control; however, they are not completely accounted for through variable-centered methods, as person-centered variations in these behaviors may co-exist independently. The present study used a person-centered, network-informed approach to explore self-control profiles latent within persons, the corresponding networks of prosocial behavior, along with responses to node perturbation.

**Methods:**

A cross-sectional sample of 1,417 early adolescents (M = 13.57 years, SD = 1.10) was recruited from junior middle schools in Guangxi, China. Using self-reported multidimensional self-control and prosocial behavior, we conducted latent profile analysis (LPA), Ising network estimation, Gaussian Graphical Model estimation, and simulation-based node-perturbation analysis (Network Intervention Response Analysis; NIRA) to identify profile-specific association patterns and candidate node-level leverage points.

**Results:**

LPA identified four self-control subgroups: Lowest, Low-to-Moderate, Moderate-to-High, and Highest. Overall prosocial behavior tended to be higher in profiles with higher self-control, although item-level patterns were not uniformly monotonic. The Low-to-Moderate self-control subgroup showed the densest pattern of associations among prosocial nodes, a pattern that may reflect stronger behavioral coupling but should not be interpreted as necessarily adaptive. Hypothetical node perturbations suggested profile-specific sensitivities: lower self-control profiles were more responsive to emotionally reactive and request-based helping nodes (e.g., PB24: Compliant helping), whereas higher self-control profiles were more responsive to non-reciprocal and principle-oriented helping nodes (e.g., PB25: Pure altruism). These patterns indicate different association structures rather than moral superiority or confirmed intervention effects.

**Conclusions:**

The present research offers person-first, network-informed evidence that self-control profiles correspond to both the extent of, and structure behind, prosocial behaviors during early adolescence. The node-perturbation results should be interpreted as hypothesis-generating evidence for potential targets requiring longitudinal and experimental validation. These findings suggest that school-based programs in the future should incorporate cautious, profile-sensitive measures instead of claims that simulating specific targets is a better method than universal target methods.

## Introduction

Ages 10–14 years is referred to as early adolescence, which is a sensitive developmental stage characterized by quick physical development and changes in social roles (Blum et al., 2014). During this period, prosocial behavior becomes especially important for peer acceptance, social competence, and positive youth development (Carlo and Randall, 2002; Crone and Achterberg, 2022). Prosocial behavior is not a single motivational category: public helping may involve social recognition, compliant helping may reflect responses to requests or perceived expectations, emotional helping is often triggered by empathic arousal, and altruistic or anonymous helping may reflect more internalized or non-reciprocal motives. Introducing these distinctions is important because the same observable helping act can arise from different developmental, emotional, and social processes.

Self-control plays a role in the way that adolescents demonstrate prosocial behaviors, because self-control allows adolescents to inhibit self-focused impulses and regulate emotional responses to events, as well as to continue focusing on the goals of helping others (Li et al., 2019; Nigg, 2017). At the same time, self-control should not be equated with moral superiority, nor should lower self-control be interpreted as an absence of prosocial motivation. Prosocial behavior is also shaped by peer norms, school climate, family socialization, cultural expectations, and opportunities for social participation. Therefore, the present study treats self-control as one regulatory context associated with prosocial behavior rather than as a deterministic cause of adolescents' prosocial or moral development.

Many earlier studies have contributed much information to the field; however, there is still a need for further research in this area due to the limitations of previous studies regarding the nature of self-control as an overarching global driver or predictor for all adolescents (Dou et al., 2019; López-Pernas et al., 2026). The person-centered method overcomes this limitation by identifying naturally occurring profiles or typologies of self-control based upon the multidimensional nature of self-control (Berlin et al., 2014; Spurk et al., 2020). The person-centered method is most applicable during the early stages of adolescence when adolescents develop their regulatory capacities, social motivations, and peer-oriented behavior at different rates from each other.

Beyond person-centered classification, network analysis allows prosocial behaviors to be examined as interconnected components rather than as interchangeable indicators of a single latent score (Borsboom et al., 2021; Epskamp et al., 2018). In the present study, LPA first identifies which self-control configurations characterize different adolescents, and network analysis then examines how specific prosocial tendencies are organized within each profile. This “who differs” and “how the system is organized” logic provides a clearer theoretical bridge between self-control heterogeneity and fine-grained prosocial behavior patterns while keeping technical model details in the Methods section.

To further explore possible candidate leverage points, we used computer-simulated node-perturbation analysis. This procedure virtually changes the activation propensity of specific prosocial nodes and estimates how such changes are associated with the rest of the fitted network. Because no real intervention was implemented, these analyses are best understood as hypothesis-generating simulations that help prioritize future longitudinal or experimental tests, not as evidence of intervention effectiveness.

Accordingly, the present study investigated early adolescents and examined the self-control–prosocial-behavior system with three objectives: (1) to identify heterogeneous latent classes of self-control using multi-faceted indicators via Latent Profile Analysis; (2) to compare levels and item-level patterns of prosocial behavior across these self-control profiles; and (3) to explore whether hypothetical node perturbations produce profile-specific changes in the fitted prosocial behavior networks. Based on prior theory and empirical evidence (De Ridder et al., 2012; Hevey, 2018; Hofmann, 2024; Masferrer et al., 2020), we examined three cautious hypotheses: (H1) early adolescents would show distinct self-control profiles characterized by different patterns of inhibitory and initiatory regulation; (H2) these profiles would differ in prosocial behavior levels and item-level tendencies; and (H3) profile-specific network simulations would suggest different candidate nodes for future intervention testing, rather than demonstrating causal cascade effects.

By examining self-control heterogeneity in relation to prosocial functioning, this study addresses the primary question: “How are distinct self-control profiles associated with the organization of prosocial behavior in early adolescence, and what candidate nodes may be worth testing in future profile-sensitive programs?” The findings are expected to provide a cautious empirical basis for developing developmentally sensitive, profile-matched school-based psychological education and behavioral intervention research.

## Materials and methods

### Research design

This study employed a cross-sectional survey design based on data collected from junior middle schools in Guangxi province, China.

### Sampling and participants

Participants were recruited using a convenience sampling strategy from 31 intact classes in selected junior middle schools. This approach was adopted because access to school-based adolescent samples was necessarily coordinated through participating schools and intact classes during regular teaching hours, making probability-based recruitment impractical in the present field setting. In developmental and school-based research, convenience sampling remains common when researchers prioritize feasibility, standardized administration, and transparent reporting of sampling boundaries (Bornstein et al., 2013; Etikan et al., 2015; Jager et al., 2017). Initially, 1,550 early adolescents aged 12–15 years were recruited. Of the 1,550 questionnaires distributed, 1,532 were returned. After rigorous data cleaning, 115 questionnaires were excluded because of extensive missing values, patterned responses (e.g., straight-lining), random answering, or incompleteness. The final analytic sample consisted of 1,417 valid responses, yielding an effective response rate of 92.49%. Because the sample was regionally bounded and recruited through a non-probability school-based procedure, generalization beyond Guangxi and similar school contexts should be made cautiously.

The final sample comprised 1,417 adolescents, including 50.25% boys and 49.75% girls. The mean age was 13.57 years (SD = 1.10). Regarding residence, 47.99% of the participants were from urban areas and 52.01% were from rural areas. In addition, 48.84% single-child adolescents and 51.16% were non-single-child adolescents. In terms of family structure, 89.56% lived in intact families, whereas 3.74% and 6.70% were from remarried and single-parent families, respectively. Detailed demographic information is presented in Table 1.

**Table 1:** Demographic characteristics by latent self-control class (*N* = 1,417).

Variable	Category	Total	Class 1	Class 2	Class 3	Class 4
Gender	Male	712 (50.25%)	82 (51.25%)	236 (48.16%)	320 (49.54%)	74 (61.16%)
	Female	705 (49.75%)	78 (48.75%)	254 (51.84%)	326 (50.46%)	47 (38.84%)
Age	—	13.57 (1.10)	13.93 (5.62)	13.64 (1.02)	13.64 (0.93)	13.73 (3.40)
Single child	Single child	692 (48.84%)	67 (41.88%)	232 (47.35%)	328 (50.77%)	65 (53.72%)
	Non-single child	725 (51.16%)	93 (58.12%)	258 (52.65%)	318 (49.23%)	56 (46.28%)
Family location	Rural	737 (52.01%)	83 (51.88%)	243 (49.59%)	351 (54.33%)	60 (49.59%)
	Urban	680 (47.99%)	77 (48.12%)	247 (50.41%)	295 (45.67%)	61 (50.41%)
Family type	Intact	1,269 (89.56%)	143 (89.38%)	439 (89.59%)	579 (89.63%)	108 (89.26%)
	Remarried	53 (3.74%)	5 (3.12%)	24 (4.90%)	19 (2.94%)	5 (4.13%)
	Single-parent	95 (6.70%)	12 (7.50%)	27 (5.51%)	48 (7.43%)	8 (6.61%)

Values are *n* (%) except for age, which is presented as M (SD). Class 1–4 denote the four latent self-control profiles identified by latent profile analysis (LPA). Gender, age, only-child status, family location, and family type distributions are presented for the total sample and for each latent class separately.

### Inclusion and exclusion criteria

To ensure the rigor and developmental relevance of the sample, explicit inclusion and exclusion criteria were established before data collection.

The inclusion criteria were as follows: (1) participants were aged between 12 and 15 years; (2) they were enrolled as full-time students in the selected junior middle schools; and (3) they participated voluntarily, with written informed consent obtained from their parents or legal guardians and assent obtained from the adolescents themselves.

The exclusion criteria were as follows: (1) a history of, or current diagnosis of, clinical psychiatric disorders (e.g., major depressive disorder, anxiety disorders, or schizophrenia); (2) current use of psychiatric medication or engagement in regular psychotherapy; (3) severe cognitive, sensory, or motor impairments that would preclude independent comprehension and completion of the questionnaires; and (4) submission of incomplete questionnaires or invalid response patterns, such as straight-lining or completing the survey in an implausibly short time (e.g., under 150 seconds). These criteria also mean that adolescents currently experiencing clinical or treatment-related self-regulation difficulties were underrepresented, which may have reduced variability in self-control and prosocial behavior.

### Procedure

Data collection was conducted during regular school hours with the assistance of classroom teachers and trained research assistants. Classroom teachers were responsible primarily for coordinating class time, maintaining routine classroom order, and facilitating communication with the schools, whereas trained research assistants administered the survey procedures and provided standardized instructions. This arrangement was adopted to minimize disruption to normal teaching activities and to ensure that data collection among minors was conducted in an orderly and developmentally appropriate school context. Before the survey began, students received standardized instructions emphasizing anonymity, confidentiality, and voluntary participation. Participants were asked to complete the questionnaire battery independently and truthfully based on their own experiences. Teachers did not participate in scoring, did not guide item responses, and were not allowed to inspect individual questionnaires. Although these procedures reduced direct teacher influence, social desirability cannot be fully ruled out in a school-based self-report setting.

The assessment battery included a demographic questionnaire, the Self-Control Ability Questionnaire (SCAQ), and the Chinese version of the Prosocial Tendencies Measure (PTM). All questionnaires were collected on site immediately after completion.

### Ethical considerations

This study was conducted in accordance with the principles of the Declaration of Helsinki (World Medical Association, 2013). The study protocol was reviewed and approved by the Human Ethics Committee of Guangxi Normal University (Approval Number: 25TYB400). Written informed consent was obtained from the parents of all participants prior to data collection, and assent was obtained from the adolescents themselves before they completed the survey.

### Sample size considerations

An adequate sample size is also essential to guarantee the reliable and stable estimating of parameters in multivariable statistical analyses, especially in psychological network models, which are sensitive to sampling variability and model complexity (Epskamp et al., 2018). Methodological work indicates that for networks with moderate to high node density, e.g. > 20 nodes, a sample size of at least 1000 participants is required in order to recover a high-quality network structure and reduce the number of spurious correlations (Epskamp and Fried, 2018; Wolf et al., 2013).

Accordingly, the final sample size of 1,417 early adolescents exceeded commonly recommended methodological thresholds. This sample size provided sufficient statistical power to detect subtle but meaningful associations between dimensions of self-control and prosocial behavior. In addition, bootstrapping-based stability analyses showed that the correlation stability (CS) coefficients of all estimated network properties were consistently above 0.50, indicating that the sample size was adequate for stable and reliable estimation of network parameters and subsequent simulated intervention analyses.

### Measures

The assessment included a demographic questionnaire, the Self-Control Ability Questionnaire (SCAQ), and the Chinese version of the Prosocial Tendencies Measure (PTM). The focal measures relevant to the present study are described below. Because both focal constructs were assessed by self-report during the same session, common-method variance and socially desirable responding were considered as interpretive limitations rather than eliminated by design.

#### Demographic characteristics

A demographic questionnaire was designed by the authors to collect participants' background information, which consisted of five domains: gender, grade level, locality (urban vs. rural), single-child status, and family type (intact family, single-parent family and remarried family). These covariates were included not only to characterize the sample but also to support a more nuanced interpretation of the associations between self-control and prosocial behavior across subpopulations. Notably, previous investigations showed that they are two of the most consistent correlates explaining individual variations in self-control and prosocial behaviors during early adolescence (Nigg, 2017; Wang et al., 2017). Demographic variables have been included to describe the sample and the latent profiles of the networks built to capture self-control and prosocial behavior. However, it is important to note that these demographic variables were not included in the demographics as covariate-adjusted predictors for network comparison, and therefore should not be used as predictors when interpreting differences between subpopulations in the analysis.

#### Measurement of self-control

Self-control was assessed using the *Self-Control Ability Questionnaire* (SCAQ), developed specifically for Chinese adolescents by Wang and Lu (2004). This instrument was selected because it captures the unique sociocultural and educational contexts of Chinese youth, demonstrating robust ecological validity compared to western-developed scales. Prior validation studies have consistently confirmed its multidimensional structure and strong psychometric properties within Chinese middle school populations (Wang and Lu, 2004). This instrument consists of 36 items designed to measure three distinct dimensions of self-control: thinking control, emotional control, and behavioral control. Participants rated items on a 5-point Likert scale. The total score ranges from 36 to 180, with higher scores indicating superior self-control capabilities. In the current study, the scale demonstrated excellent internal consistency (Cronbach's α = 0.90).

#### Measurement of prosocial behavior

Prosocial behavior was measured using the Chinese version of the *Prosocial Tendencies Measure (PTM)*, originally developed by Carlo and Randall (Carlo and Randall, 2002) and culturally adapted and revised by Kou et al. (2007). We selected this specific revised version because it accounts for collectivist cultural nuances—such as the emphasis on social harmony and interpersonal relationships—and has demonstrated excellent construct and convergent validity across numerous studies involving Chinese adolescents (Kou et al., 2007). The inventory comprises 26 items distributed across six subscales: public, anonymous, altruistic, compliant, emotional, and dire prosocial tendencies. Responses were collected on a 5-point Likert scale ranging from 1 (“does not describe me at all”) to 5 (“describes me very well”). To ensure clarity and completeness, the internal consistency reliability (Cronbach's α) for the overall scale in this study was reported as 0.87, indicating high reliability.

### Data analysis

Descriptive statistics were used to summarize demographic characteristics (e.g., gender, grade, residence, only-child status, and family structure) across profiles. Differences in prosocial behavior scores across latent profiles were then examined using non-parametric tests (Kruskal–Wallis *H* tests), followed by appropriate *post-hoc* comparisons when necessary.

#### Latent profile analysis

To identify heterogeneous subgroups characterizing adolescents' self-control patterns, Latent Profile Analysis (LPA) was conducted using Mplus 7.0. The determination of the optimal number of profiles relied on an integration of multiple fit indices, as no single metric is definitive. These indices included the Akaike Information Criterion (AIC), Bayesian Information Criterion (BIC), and Sample-Size Adjusted BIC (SABIC), with lower values indicating superior model fit (Nylund et al., 2007). In addition, the Bootstrapped Likelihood Ratio Test (BLRT) was used to compare models with k profiles to those with k-1 profiles. A significant *p* value indicates that the model with a greater number of profiles provides a significantly better fit to the data. Taking into account statistical fit indices, significance tests, and the interpretability and size of the profiles, a four-profile solution was selected as the optimal model. All identified profiles contained an adequate proportion of participants and were theoretically interpretable.

Classification accuracy was evaluated using Entropy, with values exceeding.80 generally considered indicative of good separation (Tein et al., 2013). To ensure model stability and prevent over-extraction, models were rejected if the smallest profile comprised less than 5% of the total sample (Spurk et al., 2020). The final selection of the optimal profile solution was grounded in a synthesis of these statistical indicators and theoretical interpretability. Following the identification of distinct profiles, differences in prosocial behavior scores across subgroups were examined using the Kruskal-Wallis *H* test with Dunn's *post-hoc* analysis, and the results were visualized in R.

#### Ising model and Gaussian graphical model (GGM) network analysis

Ising models and Gaussian Graphical Models (GGMs) were used to model the conditional dependence structure among prosocial behavior items, which endow with complementary insights within discrete and continuous way of modeling the data.

Before estimating the Ising networks, the initial Likert-scale scores of the 26 prosocial behavior items were dichotomized for binary network estimation (0 = relatively inactive; 1 = relatively active). Specifically, median thresholding was used: responses greater than or equal to the sample median on a given item were coded as relatively active (=1), whereas responses below this value were coded as relatively inactive (=0). This coding indicates relative activation within the present sample and should not be interpreted as a clinical, moral, or categorical classification of adolescents' prosocial tendencies.

The use of median-based dichotomization was adopted to support Ising model estimation and to maintain sufficient variability across nodes, particularly when modeling co-activation patterns in binary data (Borsboom, 2017; Epskamp and Fried, 2018). Nevertheless, dichotomizing Likert-scale responses can reduce information, compress individual variability, and simplify the complexity of adolescent prosocial behavior. Therefore, we also estimated Gaussian Graphical Models based on the original continuous item scores as a complementary sensitivity-oriented approach.

Although prior network-psychometric work suggests that quantile-based cut-points can preserve broad network structure under some conditions (Epskamp and Fried, 2018; van Borkulo et al., 2014), this remains a debated methodological decision. For this reason, results based on the Ising models are interpreted cautiously and in conjunction with the continuous GGM results rather than as definitive evidence of naturally discrete prosocial states.

Supplementing this Ising model framework and preserving information from responses to individual items on the continuous scale, Gaussian Graphical Models (GGMs) were built based on the raw Likert-scale scores. GGMs were estimated using EBIC improvement for the glasso procedure, resulting in sparse partial correlation networks where edges represent linear relationships between nodes once other variables have been controlled for. By studying both Ising and GGM networks, we could investigate the invariance of network structure from discrete to continuous representations.

Full sample network estimation was performed as well as for each of the specific latent self-control profiles. To allow for cross-network comparison, we used the same layout algorithm across all visualizations. Expected Influence (EI) was used to measure the importance of nodes, considering magnitude and sign for connecting edges, in terms of who is influencing a network most overall.

#### Simulated node-perturbation analysis

To avoid implying a real-world intervention, NIRA was understood as a way of estimating how changes in one area of an Ising network (node perturbation) affect activation of that network, without actually implementing that change. NIRA utilized an Ising model and assessed the associations between hypothetical alterations to each node's activation propensity and the total activation of the Ising Network (Epskamp et al., 2018). In the present study, the Ising networks included 26 prosocial behavior items (qsh1–qsh26) as nodes. Self-control variables were not modeled as network nodes but were used to define latent self-control profiles via LPA. NIRA analyses were conducted for the full sample and separately within each self-control profile.

Using the estimated Ising model parameters, NIRA generated 5,000 simulated datasets for each of the network nodes. Each simulated dataset had a baseline condition with default parameters and also contained specific conditions in which the nodes‘ threshold parameters were shifted up and down by ±2 standard deviations to simulate decreases or increases in the nodes' activation propensities. The number of active nodes in the prosocial behavior network (network sum score) under each of the simulated datasets was then also summarized.

The Ising model threshold parameters represent nodes' propensity for activation, and the estimations of threshold parameters are generated through a series of logistic regression analyses, where the intercept of the logistic equation for each node represents the actual node's threshold (Epskamp, 2020). Since we used self-control to develop latent profiles of self-control, the results of the network sum score refer to the four nodes that are identified as being 'active' when using the Ising network model parameters and not as a measure of self-control across all nodes. Therefore, cross-sectional NIRA results serve as potential hypotheses to further test rather than concluding a cause and effect relationship between self-control and other behaviors.

## Results

### Sample characteristics and descriptive statistics

A total of 1,417 early adolescents were included in the analyses, with 50.25% identifying as male and 49.75% as female. The mean age of the sample was 13.57 years (SD = 1.10). Within the latent self-control profiles, the Class 4 group showed the highest proportion of male students (61.16%), whereas the Class 2 group had the highest proportion of female students (51.84%). Regarding family structure, the sample was predominantly composed of adolescents from intact families (89.56%), with smaller representations from single-parent (6.70%) and remarried families (3.74%). Additionally, 48.84% of the participants were only children. Class 1 was characterized by the highest percentage of non-only children (58.12%), while Class 4 presented the highest percentage of only children (53.72%; Table 1). Means and standard deviations of all PB items across the self-control profiles are presented in Table 2.

**Table 2:** Means and standard deviations of PB items across latent self-control classes.

Variable	Class 1	Class 2	Class 3	Class 4	Total
PB 1	4.03 (0.95)	3.95 (0.94)	4.02 (0.93)	4.17 (0.86)	4.01 (0.93)
PB 2	4.24 (1.01)	4.18 (0.99)	4.29 (0.92)	4.35 (0.83)	4.25 (0.95)
PB 3	4.11 (1.07)	4.09 (0.97)	4.15 (0.97)	4.23 (0.95)	4.13 (0.98)
PB 4	3.63 (1.30)	3.57 (1.10)	3.58 (1.15)	3.70 (1.15)	3.59 (1.15)
PB 5	4.37 (0.89)	4.19 (0.92)	4.35 (0.85)	4.42 (0.82)	4.30 (0.88)
PB 6	3.76 (1.16)	3.68 (1.02)	3.75 (1.06)	4.02 (1.07)	3.75 (1.06)
PB 7	3.82 (1.18)	3.83 (0.95)	3.83 (1.05)	4.20 (0.89)	3.86 (1.02)
PB 8	3.86 (1.20)	3.92 (1.03)	3.98 (1.05)	4.18 (1.07)	3.96 (1.06)
PB 9	4.00 (1.11)	3.95 (1.05)	4.12 (0.98)	4.33 (0.87)	4.06 (1.02)
PB 10	4.42 (0.86)	4.38 (0.94)	4.37 (0.95)	4.51 (0.82)	4.39 (0.93)
PB 11	3.75 (1.13)	3.82 (0.99)	3.85 (0.99)	4.16 (0.92)	3.86 (1.01)
PB 12	3.91 (1.12)	4.03 (0.95)	4.06 (0.98)	4.31 (0.85)	4.05 (0.98)
PB 13	3.94 (1.19)	3.94 (1.04)	4.03 (1.02)	4.26 (0.84)	4.01 (1.04)
PB 14	3.55 (1.26)	3.67 (1.10)	3.77 (1.09)	3.86 (1.14)	3.72 (1.12)
PB 15	4.22 (0.96)	4.23 (0.93)	4.30 (0.86)	4.26 (0.85)	4.26 (0.90)
PB 16	3.96 (1.11)	4.03 (0.99)	4.08 (0.97)	4.29 (0.89)	4.06 (0.99)
PB 17	4.14 (0.98)	4.22 (0.94)	4.28 (0.95)	4.40 (0.81)	4.25 (0.94)
PB 18	4.00 (1.08)	4.03 (0.96)	4.14 (0.98)	4.30 (0.89)	4.10 (0.98)
PB 19	3.81 (1.06)	4.01 (0.96)	3.96 (0.97)	4.17 (0.92)	3.98 (0.97)
PB 20	3.71 (1.25)	3.74 (1.07)	3.89 (1.05)	4.15 (1.01)	3.84 (1.08)
PB 21	3.49 (1.37)	3.59 (1.16)	3.71 (1.18)	4.02 (1.04)	3.67 (1.19)
PB 22	3.99 (4.25)	3.83 (1.09)	3.91 (1.10)	4.01 (1.02)	3.90 (1.75)
PB 23	4.32 (0.97)	4.27 (0.94)	4.34 (0.88)	4.45 (0.84)	4.32 (0.91)
PB 24	4.24 (0.89)	4.25 (0.89)	4.27 (0.89)	4.39 (0.78)	4.27 (0.88)
PB 25	4.14 (0.99)	4.14 (1.00)	4.20 (0.95)	4.40 (0.89)	4.19 (0.97)
PB 26	4.11 (1.10)	4.09 (1.00)	4.23 (0.98)	4.36 (0.87)	4.18 (0.99)

Values are presented as mean (standard deviation). PB, prosocial behavior items. Class 1–4 denote the four latent self-control profiles identified by latent profile analysis (LPA).

Overall, prosocial behavior scores were highest in Class 4 (high self-control profile) and lowest in Class 1 (low self-control profile), with the other groups falling between the two. Across the full sample, items PB10 (*M* = 4.39, *SD* = 0.93), PB23 (*M* = 4.32, *SD* = 0.91), PB5 (*M* = 4.30, *SD* = 0.88), PB24 (*M* = 4.27, *SD* = 0.88), and PB15 (*M* = 4.26, *SD* = 0.90) exhibited the highest mean values, indicating that these specific behaviors occupy prominent positions within the broader network of prosocial tendencies. Differences between Class 1 and Class 4 were marked for most items—particularly PB21 (*M*_*diff*_ = 0.53), PB11 (*M*_*diff*_ = 0.41), and PB7 (*M*_*diff*_ = 0.38)—reinforcing the positive association between self-control level and the endorsement of prosocial behaviors.

### Latent profile analysis of self-control and associated prosocial behavior symptom patterns

The outcomes from latent profile analysis endorse a four-profile approach for managing self-control levels. While a model with five profiles may provide lower numerical estimates of information criteria than a four-profile model, class assignment was not determined strictly via fit statistics. A fifth profile typically divides a clear, i.e., adjacent, grouping into two without providing an additional theoretical framework or structure. A 4-profile approach yielded satisfactory class sizes, distinct separations among groups, and increased clarity in developing an interpretative significance from the data. Having stated the above-mentioned criteria collectively, the four-profile approach offers the greatest likelihood of providing the simplest and most meaningful theoretical explanations.

As shown in Table 3, distinct and systematic gradients in prosocial behavior emerged across the self-control profiles. Overall, Class 4 exhibited the highest mean scores across nearly all prosocial behavior items, whereas Class 1 consistently showed the lowest levels. Classes 2 and 3 displayed intermediate patterns, forming a monotonic increase in prosocial behavior as self-control levels increased.

**Table 3:** Latent profile analysis models and statistical fit indices.

Model	AIC	BIC	aBIC	Entropy	LMR_p	BLRT_p
1-class	13,286.524	13,318.062	13,299.002	NA	NA	NA
2-class	12,358.646	12,411.209	12,379.442	0.69	0.0066	0
3-class	11,367.835	11,441.423	11,396.95	0.862	1e-04	0
4-class	10,603.006	10,697.619	10,640.44	0.894	0.128	0
5-class	9,938.176	10,053.814	9,983.928	0.919	0	0

AIC, akaike information criterion; BIC, Bayesian information criterion; aBIC, sample-size adjusted BIC; LMR, Lo–Mendell–Rubin likelihood ratio test.

At the item level, particularly pronounced mean differences between Class 1 and Class 4 were observed for several prosocial behavior items, including PB21, PB11, and PB7. For these items, adolescents in the highest self-control profile demonstrated substantially higher average scores compared with those in the lowest self-control profile, with intermediate profiles showing graded transitions between the two extremes. These patterns suggest that variations in self-control are associated not only with overall levels of prosocial behavior but also with the intensity of specific prosocial actions.

**Figure 1:**
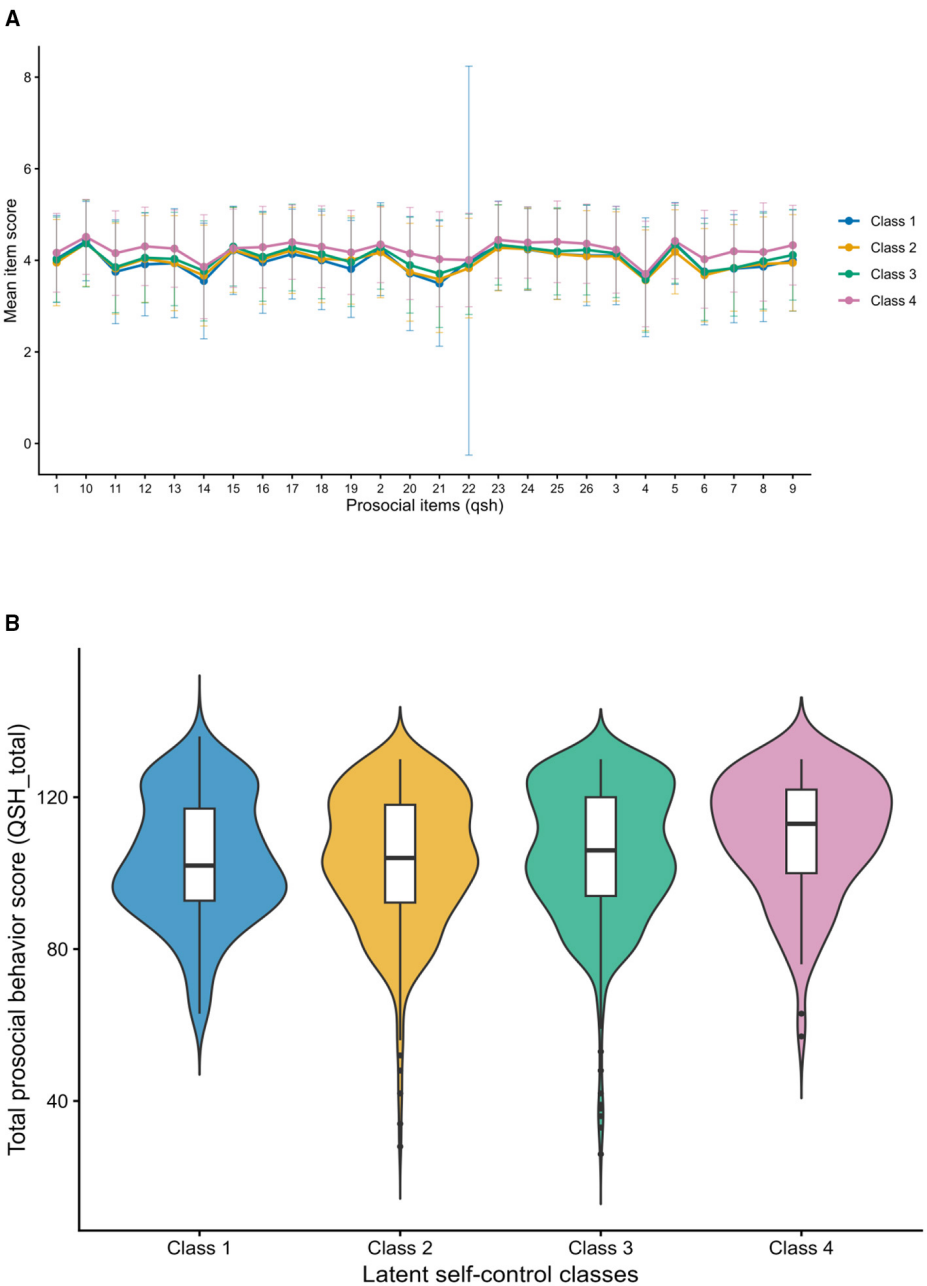
**(A)** mean prosocial behavior item scores across four latent self-control classes. **(B)** Distribution of total prosocial behavior item scores across four latent self-control classes.

Our results suggest that self-control profiles are associated with differences in the number and component-level patterns of prosocial behaviors. Because of our use of a cross-sectional study design, these results should be interpreted as evidence that there is a specific association between self-control profiles and prosocial behaviors, rather than an indication of the causal influence of self-control on the multiple, different patterns of prosocial behavior (Figure 1).

### Ising network structures of prosocial behavior items

**Figure 2:**
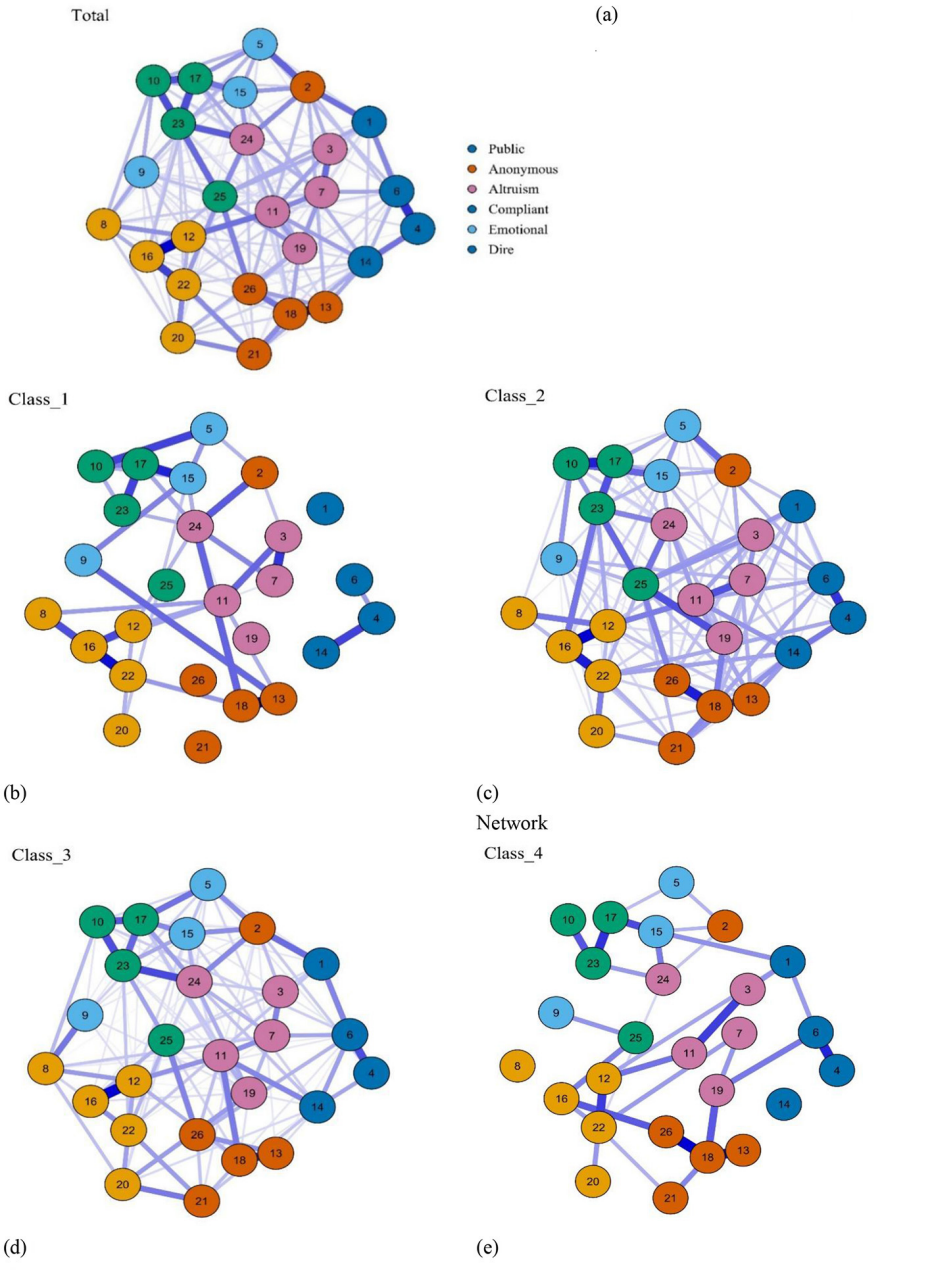
Ising network structures of prosocial behavior symptoms across the full sample and latent self-control profiles in early adolescents. **(a)** Total: full sample network. **(b)** Class_1: lowest self-control prosocial network. **(c)** Class_2: low-to-moderate self-control prosocial network. **(d)** Class_3: Moderate-to-high self-control prosocial network. **(e)** Class_4: highest self-control prosocial network.

The Ising network structures of prosocial behavior items are presented in Figure 2 for the full sample and for the four latent self-control profiles identified by Latent Profile Analysis (LPA). The 26 prosocial behavior items are grouped into six domains: Public (blue-green), Anonymous (orange), Altruism (pink), Compliant (dark blue), Emotional (light blue), and Dire (green). In the figure, blue edges represent positive associations, and thicker, more saturated edges indicate stronger associations. Across the five networks, most estimated edges were positive.

The full-sample network showed relatively dense overall connectivity. At the subgroup level, the four self-control profiles displayed different association patterns. Visually, the Low-to-Moderate Self-Control profile (Class 2) showed the densest network, with multiple within-domain and cross-domain edges among prosocial items. This density indicates stronger conditional coupling among prosocial tendencies within the fitted network, but it should not be equated with adaptiveness.

The Lowest Self-Control prosocial network (Class 1) also showed several within-domain connections, particularly among altruism-, public-, and dire-related items, but cross-domain edges were fewer than in Class 2. The Moderate to High and Highest Self-Control Groups exhibited progressively sparser connections despite having also reported higher average prosocial scores, suggesting that even though an individual scores higher in support of prosocial Behaviors, does not mean the individual is necessarily coupled to the behaviors of all prosocial items.

Overall, Figure 2 shows variation in the density and distribution of edges across the self-control profiles. While this information provides evidence for how each self-control profile organizes prosocial Behaviors differently, it is important to interpret the visual data carefully, as the density of a network may indicate coupling, rigidity, common method variance or differential variance across the same group. Comparison of formal networks (where available) should also be taken into consideration for more robust interpretations of the visual representations of network connectivity.

### Expected influence centrality and network stability

To identify nodes with relatively high centrality within the fitted networks, we computed Expected Influence (EI). EI incorporates the magnitude and sign of edges incident to a node and indicates how strongly a node is statistically connected to other nodes in the estimated network. EI should not be interpreted as evidence that a node causally drives the network.

**Figure 3:**
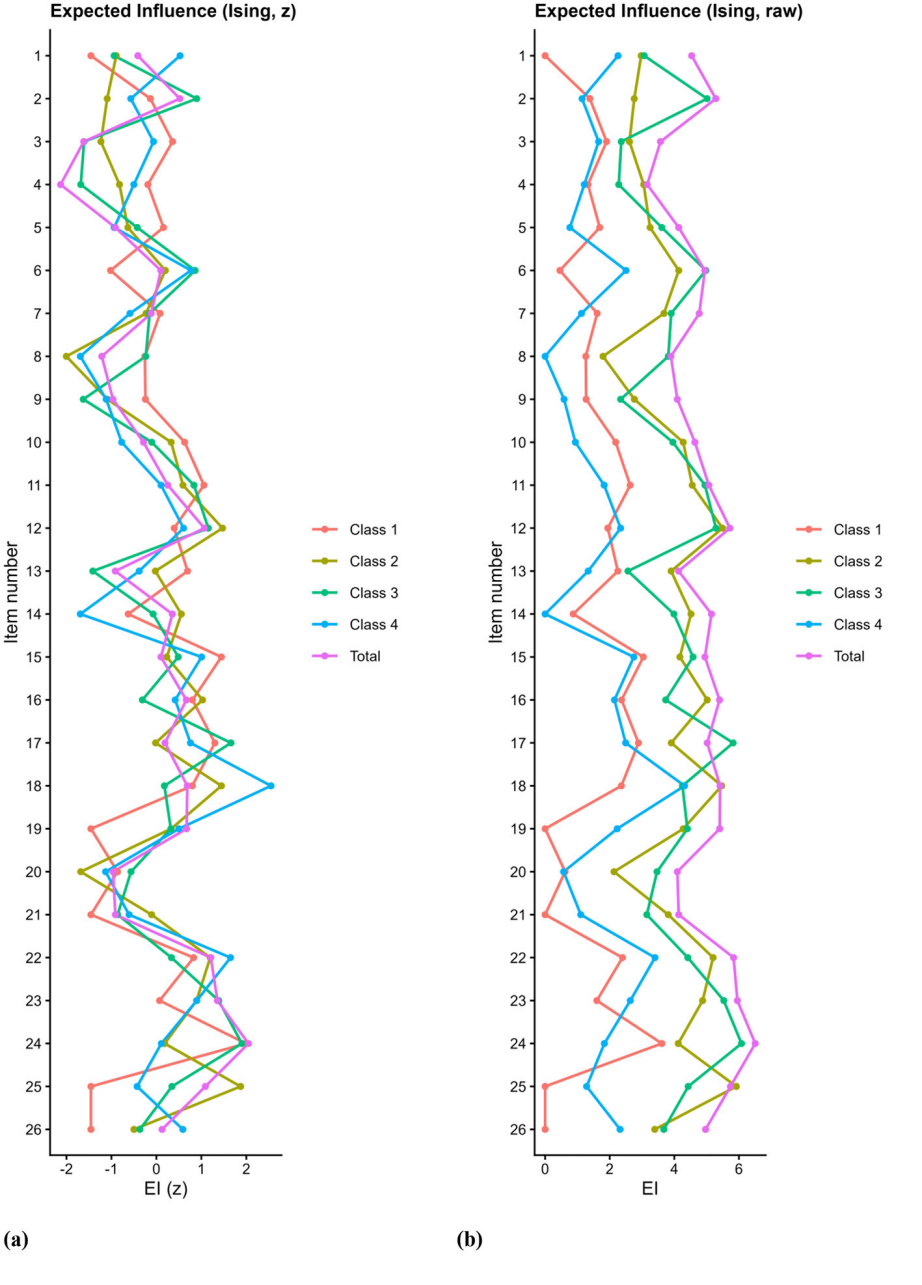
Expected influence centrality of prosocial behavior items in Ising networks across latent self-control profiles. **(a)** Standardized expected influence centrality across prosocial behavior items. **(b)** Raw expected influence centrality across prosocial behavior items.

As shown in Figure 3, several prosocial behavior items showed consistently high EI values in the full-sample Ising network. Emotion-based prosocial behaviors (e.g., Item 2 and Item 21) and norm- or request-responsive behaviors (e.g., Item 3, Item 7, Item 11, and Item 24) were relatively central. These patterns are developmentally meaningful because everyday emotional responsiveness and request-based helping are common forms of adolescent prosocial behavior.

These high-EI nodes were broadly connected to other prosocial behaviors, suggesting that they functioned as central association points or bridging elements within the estimated network. In contrast, prosocial behaviors involving public or emergency contexts showed comparatively lower expected influence, indicating weaker conditional connectivity in the present cross-sectional data.

Overall, these findings indicate that everyday, emotionally responsive, and norm-congruent behaviors were more central within the observed prosocial behavior network than highly salient or costly helping acts. However, centrality does not establish causal importance; these nodes are best treated as theoretically informative candidates for future longitudinal or experimental testing.

### Simulated node-perturbation analysis for the full sample

**Figure 4:**
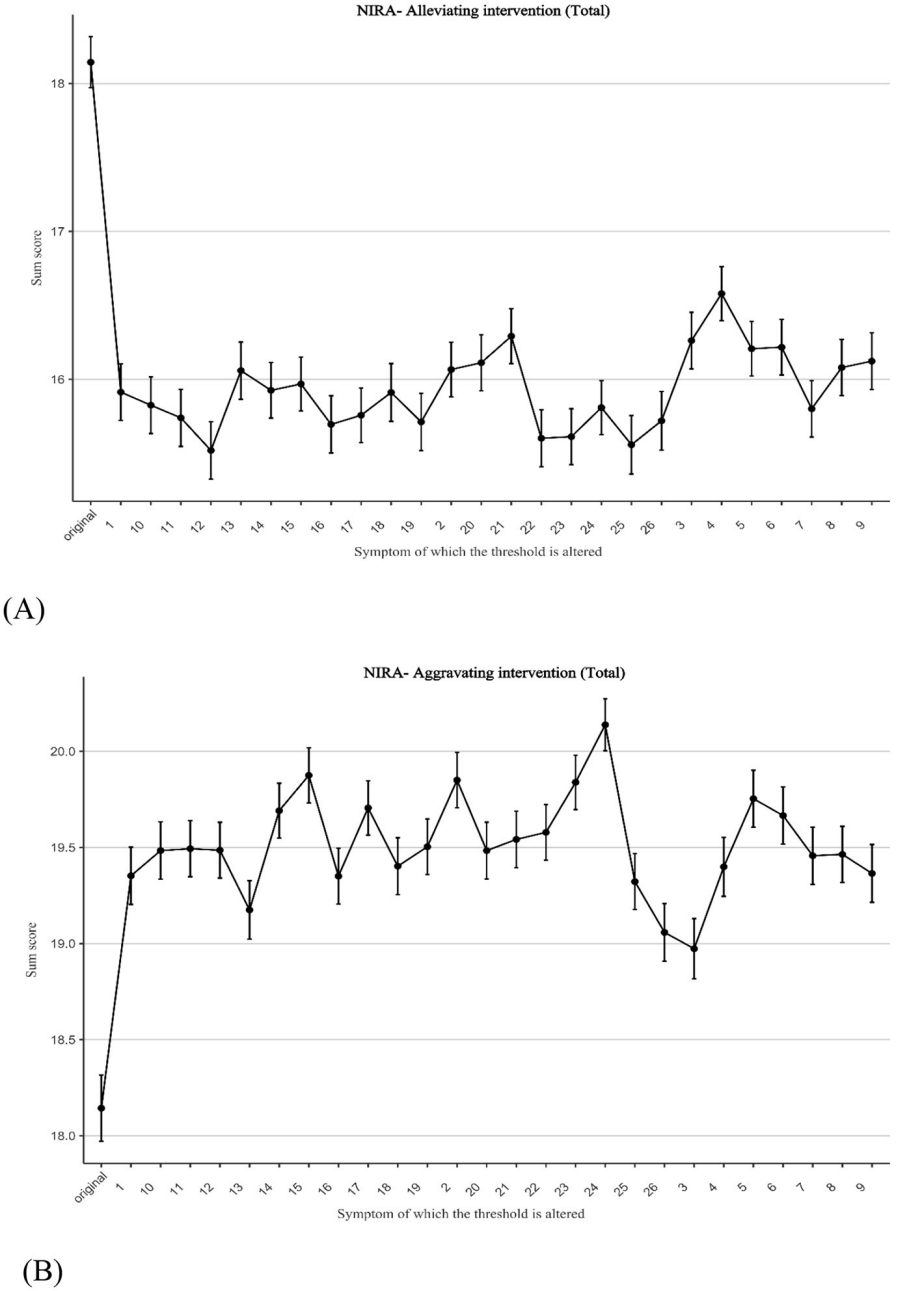
NIRA simulated node-perturbation results for prosocial behavior symptoms in the full sample (downward- vs. upward-threshold perturbations). **(A)** NIRA – downward-threshold node perturbation for the full sample. **(B)** NIRA – upward-threshold node perturbation for the full sample.

Figure 4 summarizes the results of the Network Intervention Response Analysis (NIRA) simulated node-perturbation analysis for the full sample. The analysis estimated how hypothetical upward or downward shifts in each node's activation propensity were associated with changes in the total number of active prosocial behavior nodes in the fitted Ising network.

**Figure 5:**
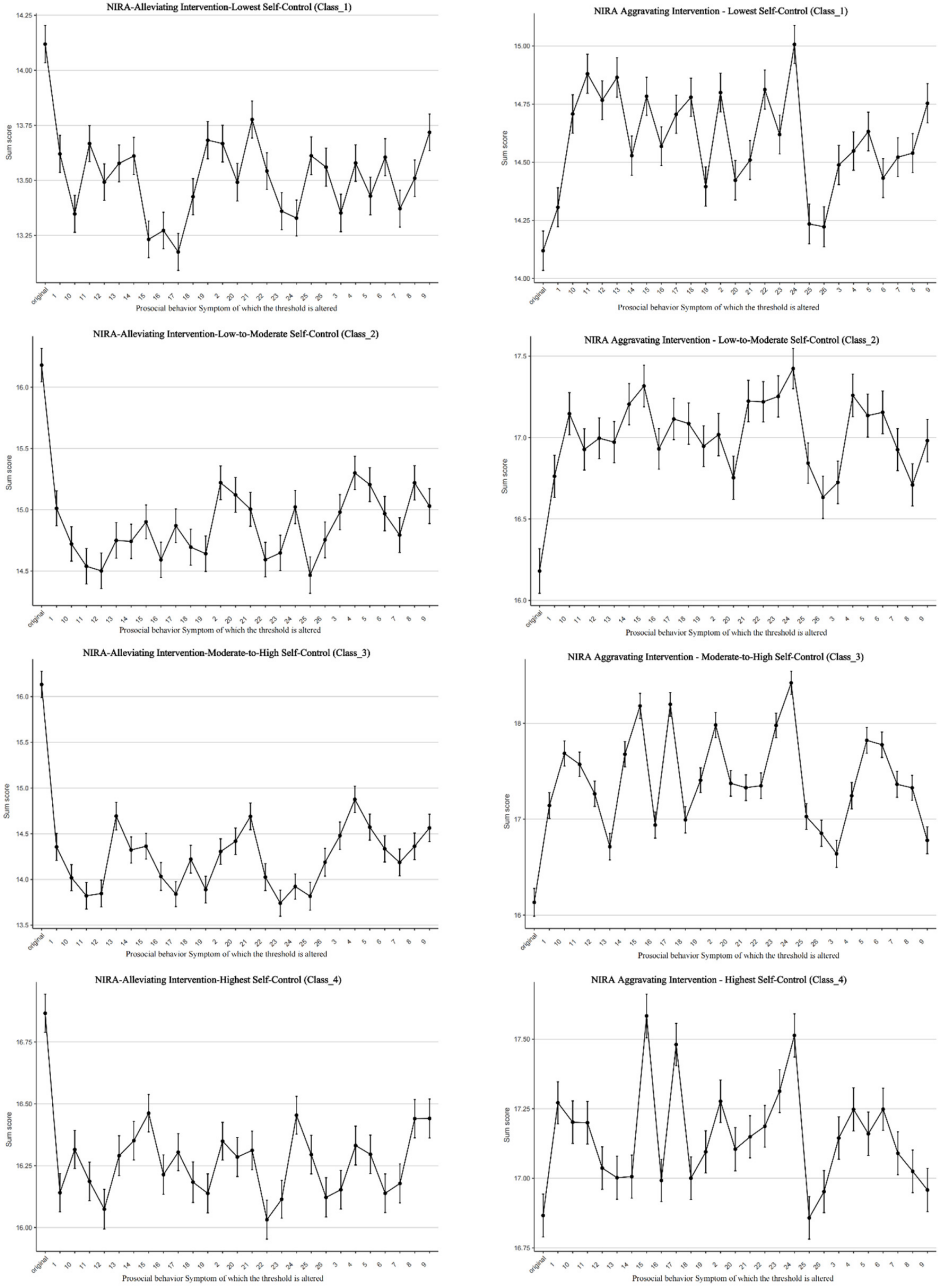
Projected effects of NIRA interventions on the prosocial behavior Ising model of four profiles.

In the downward-threshold perturbation condition (Figure 5), the activation propensity of a selected node was increased within the fitted model, allowing us to examine whether easier activation of that node was associated with broader prosocial-node activation. In the upward-threshold perturbation condition (Figure 5), the activation propensity of a selected node was reduced, allowing us to examine whether reduced activation was associated with lower overall prosocial-node activation.

These perturbation effects are model-based projections rather than empirical intervention outcomes. Therefore, nodes with larger simulated effects are described as candidate leverage points within the observed association structure, not as confirmed intervention targets.

In summary, NIRA provided insight into the relationships among multiple nodes to assist in determining which nodes exhibit the greatest degree of perturbation effects for the full data set and guide the selection of nodes to be examined in future studies regarding how to best support and facilitate the development of prosocial behaviors and, due to the predicted low effect size on changing the behaviors, the future impact of changing those behaviors and the related benefits for society through less crime and drug use, better health and higher productivity through higher civic engagement, employment and less reliance on government programmes.

### Simulated node-perturbation analysis across self-control profiles

To identify prosocial behavior items that may warrant future intervention testing, simulation-based node-perturbation analyses were conducted using the NIRA framework. Separate Ising networks were estimated for each latent self-control profile, and each prosocial behavior item was perturbed within its corresponding profile-specific network. The resulting changes in total prosocial-node activation were interpreted as model-based sensitivity patterns rather than evidence of real-world intervention efficacy.

#### Class 1 (lowest self-control)

For Class 1, upward-threshold perturbations identified PB24, PB11, and PB13 as nodes with relatively large modeled effects. Specifically, changing the activation propensity of PB24 (“When others ask me for help, I do my best to help them”), PB11 (“When others ask me for help, I quickly put aside what I am doing to help them”), and PB13 was associated with notable changes in overall prosocial-node activation. These request-based and situational helping items may reflect both genuine concern and responsiveness to social expectations; therefore, they should be interpreted cautiously rather than treated as pure altruistic indicators.

In contrast, downward-threshold perturbations showed relatively large modeled changes for PB17 and PB15. Changes in PB17 (“The time and energy I devote to volunteer service are not for gaining more rewards”) and PB15 (“When others are suffering from hunger or cold, I naturally offer them help”) were associated with broader changes in the fitted network, suggesting candidate nodes for future hypothesis testing in adolescents with the lowest self-control profile.

#### Class 2 (low-to-moderate self-control)

In Class 2, upward-threshold perturbation effects were primarily associated with PB24 and PB15. Changes in PB24 (“When others ask me for help, I do my best to help them”) and PB15 (“When others are suffering from hunger or cold, I naturally offer them help”) produced the largest modeled changes in total prosocial-node activation, indicating that request-based and emotionally responsive helping were highly coupled with the broader network.

Downward-threshold perturbations highlighted PB15, PB12, and PB11 as nodes with relatively large modeled effects. Lowering thresholds for PB15, PB12 (“I tend to help people in need without leaving my name”), and PB11 (“When others ask me for help, I quickly put aside what I am doing to help them”) was associated with broader simulated changes in the fitted network.

#### Class 3 (moderate-to-high self-control)

Class 3 demonstrated a more differentiated response surface. Upward-threshold perturbations identified PB24, PB15, and PB17 as nodes with relatively large modeled effects. Changes in these nodes were associated with notable changes in total prosocial-node activation, suggesting that both request-responsive and non-reciprocal helping were central to the fitted network for this profile.

In contrast, downward-threshold perturbation effects were strongest for PB23 and PB25. Reducing thresholds for PB23 (“I help others not for the sake of receiving help from them in the future”) and PB25 (“I often help others even if I gain no benefits from doing so”) produced the largest modeled changes, suggesting candidate principle-oriented nodes for future testing.

#### Class 4 (highest self-control)

In Class 4, upward-threshold perturbations primarily involved PB15, PB25, and PB17. Changes in PB15 (“When others are suffering from hunger or cold, I naturally offer them help”), PB25 (“I often help others even if I gain no benefits from doing so”), and PB17 (“The time and energy I devote to volunteer service are not for gaining more rewards”) were associated with the largest modeled changes in this relatively sparse network.

Downward-threshold perturbations identified PB22 and PB12 as nodes with relatively large modeled effects. Changes in PB22 (“I often make donations without others knowing, because it makes me feel good”) and PB12 (“I tend to help people in need without leaving my name”) were associated with broader simulated changes, but these findings remain model-based and require empirical validation.

#### Cross-class trends in simulated node-perturbation effects

Across classes, several systematic trends emerged. PB15 (humanitarian helping) and PB24 (immediate compliance with help requests) repeatedly appeared among nodes with relatively large modeled perturbation effects, especially in Classes 2–4. PB24 should be interpreted carefully because compliant helping may reflect both prosocial responsiveness and perceived social pressure. In higher self-control profiles, non-reciprocal or principle-oriented items such as PB23 and PB25 became more salient, but this pattern should not be read as evidence that high self-control is morally superior; rather, it suggests a different association structure among self-control profiles.

Importantly, the magnitude and shape of response curves differed across classes. Lower self-control groups showed broader response patterns, whereas higher self-control groups showed more selective sensitivity and flatter curves. Alternative explanations, including lower variance, ceiling effects, or profile-specific measurement properties, should be considered before interpreting flatter curves as resilience. These results therefore generate hypotheses about profile-sensitive node selection rather than confirming effective intervention modules.

## Discussion

The present study extends prior work on self-control and prosocial behavior by showing that self-control heterogeneity is associated not only with mean prosocial levels but also with the organization of prosocial behavior. Using a person-centered framework that combined latent profile analysis, network modeling, and simulated node perturbations, we found that prosocial behavior increased across self-control profiles at the mean level, whereas network connectivity followed a non-linear pattern. Because the present data are cross-sectional and the node perturbations are computational, these findings should be interpreted as association-based and hypothesis-generating rather than as evidence of causal developmental mechanisms or intervention effects.

### Self-control heterogeneity and mean-level differences

Given the role of self-control in supporting social adjustment, the present study adopted a person-centered framework integrating LPA, network modeling, and simulation-based analyses to examine profile-specific association patterns between self-control and prosocial behavior (Berlin et al., 2014; Borsboom et al., 2021; Epskamp et al., 2018). Profiles of self-control should be differentiated from each other when considering regulatory patterns; however, developmental context is equally important. Other social factors such as peer relationships, school environment, family socialization, warmth of parenting and culture can influence the regulation of one's behavior with regard to self-control and prosocial behavior, and therefore do not allow us to fully interpret the results from an ecological and causal standpoint.

Supporting Hypotheses 1 and 2, adolescents differed not only in overall self-control but also in the prosocial tendencies associated with these configurations. The monotonic gradient in prosocial behavior is consistent with prior work linking self-control with helping-related behavior and social adjustment (Carlo and Randall, 2002; Crone and Achterberg, 2022; Li et al., 2019). However, different prosocial forms have different motivational meanings: compliant helping may reflect responsiveness to requests or social expectations, emotional helping may reflect empathic arousal, and altruistic or anonymous helping may reflect non-reciprocal motives. Thus, higher self-control should be interpreted as associated with different prosocial profiles, not as evidence of moral superiority or a more intrinsically virtuous system.

### Non-linear prosocial network organization across self-control profiles

Beyond mean-level differences, the subgroup networks indicated that prosocial behavior was organized differently across self-control profiles. The Low-to-Moderate Self-Control group showed denser cross-domain associations, whereas the Highest Self-Control group showed a sparser pattern despite higher mean prosocial levels. In network terms, density refers to the degree to which prosocial items remain conditionally associated after accounting for other items. Dense connectivity can reflect integration among behaviors, but it may also indicate rigidity, overdependence, shared method variance, or vulnerability to cascading changes. Therefore, dense networks should not be automatically interpreted as adaptive.

One tentative interpretation is that higher self-control may be associated with more differentiated or less cross-domain-dependent prosocial tendencies. Here, “less dependent” means that endorsement of one prosocial tendency is less strongly tied to endorsement of many other tendencies in the fitted cross-sectional network. This interpretation is compatible with work suggesting that effective self-control may rely on stable, goal-congruent habits rather than constant effortful inhibition (Galla and Duckworth, 2015), but alternative explanations such as subgroup differences in variance, ceiling effects, or measurement properties remain plausible.

This pattern is theoretically meaningful because it separates behavioral level from structural organization. Adolescents with higher self-control may report stronger prosocial tendencies overall while showing weaker statistical coupling among different forms of helping. Conversely, adolescents with moderate self-control may show stronger co-occurrence among prosocial tendencies, meaning that one type of helping tends to be linked with several others. This distinction illustrates the added value of combining person-centered and network-informed approaches while avoiding claims about within-person developmental transitions that the present data cannot establish (Borsboom et al., 2021; Costantini et al., 2019).

### Profile-specific sensitivity in simulated networks

The simulated node-perturbation, considered the need for additional, hypothesis-generating information, revealed the sensitivity of candidate leverage nodes differs between profiles: individuals representing the group of Lower Self Control had a greater sensitivity to the Nodes that help those emotionally reactive and who use request-based Helping behavior than Individuals in the Higher Self Control profile, who had a greater sensitivity to the Non-Reciprocal Helping nodes and the Principle-Oriented Helping nodes. These findings are in keeping with the Multidimensional Perspective regarding Prosocial Development during Adolescence, which posits as Individual's Develop, their motives for Helping, the context or situations in which Helping occurs, and their ways of Helping become increasingly diverse (Carlo and Padilla-Walker, 2020; Crone and Achterberg, 2022). However, it is essential to note that this information is not to be viewed as establishing a moral hierarchy but simply refers to varying modeling associations between groups, rather than establishing a profile as an overall measure of Moral Maturity.

Importantly, these simulation results should be interpreted as identifying candidate leverage points within the observed association structure rather than demonstrating that manipulating a given node would necessarily produce downstream behavioral change. This distinction is especially important because cross-sectional centrality and node-perturbation findings can be unstable and are limited in their ability to identify causal targets (Rodebaugh et al., 2018). Therefore, differences in the measures across profiles provide valuable hypotheses for future longitudinal, experimental, and/or school-based intervention studies, but they do not provide strong evidence for the effectiveness of the proposed interventions.

### Integrative interpretation

In summary, the data indicate that self-control influences both the number of prosocial behaviors adolescents report as well as the degree to which the tendency to engage in prosocial behaviors co-occurs in a larger framework of behavior. This is an exploratory theme for future research. Future studies should investigate whether the same results exist when peer relationships, school climate, parenting practices, academic strains, cultural expectations, and online social environments are taken into account, as all of these contextual factors affect both the self-regulatory processes of adolescents and the motivating factors for adolescent prosocial behaviors.

## Limitations

This study still has several limitations. First, the cross-sectional design precludes strong causal inferences regarding the relationship between self-control and prosocial behavior. The estimated networks reflect between-person association structures at a single time point and cannot capture temporal dynamics, developmental transitions, or within-person change (Epskamp et al., 2018). Relatedly, centrality and node-perturbation results from cross-sectional networks may be unstable and should not be treated as definitive causal targets. Second, all focal variables were measured using adolescent self-report questionnaires administered in the same school-based session. This design may introduce common method variance and social desirability bias, especially because self-control and prosocial behavior are socially valued constructs (Padilla-Walker et al., 2016). Third, dichotomizing Likert-scale responses for Ising model estimation may have reduced variability and simplified the complexity of prosocial tendencies, even though continuous GGMs were also estimated as a complementary approach. Fourth, although demographic characteristics were described, the present network comparisons did not formally model potentially important contextual factors, including gender, family socioeconomic status, parenting style, peer relationships, school climate, academic stress, cultural expectations, and digital or social media environments. Fifth, as participants were recruited from junior middle schools in Guangxi, China, the generalizability of the findings to other regions, cultural contexts, or developmental stages remains uncertain. Finally, the simulated node perturbations are model-based projections rather than experimentally validated intervention effects; whether changing these candidate nodes produces real behavioral change must be established through longitudinal and intervention-based research.

### Implications of the study

The implications of this study include both theoretical considerations as well as practical applications. From a theoretical perspective, this study's use of latent profile analysis, network analysis, and simulation through node-perturbation modeling extends the work surrounding self-regulation and prosocial behaviors beyond merely describing mean levels of these variables to document how the profiles Type of regulating vary across types of prosocial behavior based on their structure. In practice, the findings from this study will inform how schools may develop their approaches (e.g., psychoeducation) and services (e.g., school counseling, behavioral programme development) by identifying what type of prosocial behaviors should be specifically assessed for on different types of self-regulatory profiles. Although these findings provide an avenue for generating new hypotheses, the results from this study cannot conclusively demonstrate that directing efforts within real-world programmes toward high levels of Emotional Intelligence or high-perturbation nodes will yield positive benefits. Furthermore, structured physical activity presents an opportunity for future research; structured physical activity may, therefore, provide a common basis for combining aspects of self-regulation and emotional responsiveness with co-operating, rule-following, and peer interactions; however, structured physical activity was not a direct intervention variable in the current study and therefore will need to be integrated into future applications with insights from individual node-level analyses and contextual support systems (e.g., teachers, parents, peers, school environment), while also taking into consideration cultural differences with regard to socializing processes among Chinese adolescents.

## Future directions

There are numerous ways future research can be approached. Longitudinal and temporal network studies should be utilized to determine whether an individual's prosocial network density fluctuations correspond with their individual personality characteristics or developmental periods, or if they simply reflect normative changes throughout adolescence. Future research must include several sources of data, or multiple informants and behavioral measures such as parental, teaching, and peer evaluations and observations, in addition to subjective evaluations made by the individual themselves, in order to eliminate bias due to response style and social desirability. Future research should also consider other contextual factors that influence adolescent behavior, such as parenting styles, peer relationships, school climates, the presence of academic stress and their socioeconomic status, as well as the values that form their cultural expectations and the effects of digital and social media on their lives. Future studies should be conducted among different genders and from different cultural backgrounds to determine if similar structures exist within prosocial networks across countries and cultures. Experimental and intervention based studies should also occur in actual school environments to determine whether specific target node level variables will lead to observable behavior in adolescents. Finally, qualitative studies may enhance our understanding of adolescents' perspectives regarding their motivations for different types of helping.

## Conclusions

In summary, the study suggests that there are several ways that variety in levels of self-control relates to the level of prosocial behavior and ways prosocial behavior is organized among individuals during early adolescence. The results suggest that adolescents with different types of self-control report significantly different average levels of prosociality and have different network structures, which includes the identification of different modeled candidate leverage nodes. This work contributes to a person focused, network-informed way of understanding prosocial behavior development while still maintaining a non-causal and exploratory nature. Understanding variations in levels of self-control, in turn, has important developmental meaning during early adolescence when self-regulation, peer relationships, family and school environments, and prosocial motives change rapidly. Research that incorporates longitudinal and experimental methods is needed to determine whether these identified candidate nodes can, in fact, serve as the basis for developing effective and culture-focused contextual supports for positive youth development.

## Data Availability

The original contributions presented in the study are included in the article/supplementary material, further inquiries can be directed to the corresponding author.
